# Visual learning in tethered bees modifies flight orientation and is impaired by epinastine

**DOI:** 10.1007/s00359-023-01623-z

**Published:** 2023-03-17

**Authors:** Norihiro Kobayashi, Yuji Hasegawa, Ryuichi Okada, Midori Sakura

**Affiliations:** 1 Department of Biology, Graduate School of Science, Kobe University, 1-1 Rokkodai-cho, Nada-ku, Kobe, Hyogo 657-8501 Japan; 2 Tokyo, Japan

**Keywords:** Honey bee, Flight simulator, Visual learning, Octopamine, Color discrimination

## Abstract

Visual-orientation learning of a tethered flying bee was investigated using a flight simulator and a novel protocol in which orientation preference toward trained visual targets was assessed in tests performed before and after appetitive conditioning. Either a blue or a green rectangle (conditioned stimulus, CS) was associated with 30% sucrose solution (unconditioned stimulus, US), whereas the other rectangle was not paired with US. Bees were tested in a closed-looped flight simulator 5 min after ten pairings of the US and CS. Conditioned bees were preferentially oriented to the CS after such training. This increase in preference for CS was maintained for 24 h, indicating the presence of long-term memory. Because the total orienting time was not altered by conditioning, conditioning did not enhance orientation activity itself but increased the relative time for orientation to CS. When 0.4 or 4 mM epinastine (an antagonist of octopamine receptors) was injected into the bee’s head 30 min prior to the experiment, both short- and long-term memory formation were significantly impaired, suggesting that octopamine, which is crucial for appetitive olfactory learning in insects, is also involved in visual orientation learning.

**Supplementary Information:**

The online version contains supplementary material available at 10.1007/s00359-023-01623-z.

## Introduction

Many animals use visual cues for navigation. The honey bee *Apis mellifera* is one of the species that employs visually guided navigation. Honey bees have a remarkable ability to learn the visual features that they experience during their foraging routes, including landmarks (Cartwright and Collett 1982, 1983; Fry and Wehner 2005; Menzel et al. 2018) and panoramic skylines (Towne and Moscrip 2008; Collett 2008; Towne et al. 2017). Visual learning in honey bees has been well investigated in a number of behavioral studies using the traditional Y-maze for free-flying bees (for review, see Horridge 2009; Giurfa 2012). Bees can recognize various types of visual stimuli, such as colors, patterns, and even complex paintings, associate a particular visual stimulus with food location and navigate to the goal using their visual memory (e.g., Srinivasan 1994; Wu et al. 2013; Horridge 2015). These studies provided a great opportunity to understand the cognitive aspects of honey bee vision (Giurfa 2012; Avarguès-Weber and Giurfa 2013). However, despite these detailed behavioral investigations, the neural basis of such visually guided orientation is largely unknown, mainly because of the difficulties in accessing the brain of free-flying individuals. As a solution, two recent studies succeeded in examining the role of biogenic amines in visual learning using visual conditioning of the proboscis extension response (PER) (Mancini et al. 2018; Vieira et al. 2018), an appetitive response elicited by the contact of sucrose solution with the antennae (Minnich 1932). In both cases, octopamine (OA) and dopamine (DA) modulated the appetitive visual learning of honey bees.

Virtual reality (VR) techniques are another way to address this issue (Schultheiss et al. 2017). Using VR, one can observe the navigational trajectories of a tethered animal in a certain visual environment by translating the animal’s movement into displacements of the visual stimulus. This setup is particularly useful for physiological experiments as the animal is tethered and stationary in the setup and allows the presentation of a stimulus more precisely than in a free-flying condition. Recently, a series of behavioral studies of honey bees in two- or three-dimensional VR conditions have reported that walking bees on a treadmill ball could learn to associate a visual stimulus with the location of a sucrose reward, similar to free-flying bees in a Y-maze (Buatois et al. 2017, 2018; Lafon et al. 2021, 2022; Geng et al. 2022). In addition, the involvement of the mushroom body in visual learning under VR conditions has been demonstrated by analyses of immediate early gene expression (Lafon et al. 2022; Geng et al. 2022). However, in all of these previous studies, the bees did not fly but walked on a treadmill, which differs from the normal foraging situation in which bees engage in flight activity.

Various types of flight simulators for tethered animals have been used to understand the mechanisms underlying various behaviors of flying insects, such as learning (Brembs and Heisenberg 2000, 2001; Liu et al. 2006), motor control (Kern and Egelhaaf 2000; Dickerson et al. 2019), and navigation (Mappes and Homberg 2004; Reppert et al. 2004; Warren et al. 2018). Visual learning in flying *Drosophila* has been intensively studied using a VR flight simulator, which is one of the most famous VR setups used in neuroethology (Heisenberg et al. 2001). In these studies, a fly was tethered to the center of a cylindrical arena displaying visual stimuli on its inner wall. These stimuli varied their position according to the yaw torque of the tethered fly, which was trained to associate some of them with a punishment provided by a heat beam pointed onto the abdomen. In this way, the fly learnt to avoid a certain visual stimulus and to fly toward a different safe stimulus (Wolf and Heisenberg 1991).

In contrast to *Drosophila*, studies on honey bee flying behavior using flight simulators are scarce. Flight simulator experiments found that tethered bees slightly raised their abdomens during flight as a response to image motion, presumably to reduce aerodynamic drag (Luu et al. 2011; Taylor et al. 2013). In our previous study, a tethered bee showed right- and left-turning responses to a slowly rotating overhead polarized light stimulus to orient its flight trajectory to a particular e-vector orientation (Kobayashi et al. 2020). These results indicate that the bee exhibits visually guided flight control behavior even under tethered conditions.

Here, we investigated the visual orientation learning of a tethered flying bee using a restricted two-dimensional VR flight simulator, wherein the yaw torque generated by the bee was translated into the sideways movement of the visual stimuli on a PC monitor in front of the bee. Using this apparatus, we examined whether the bee’s color preference changed before and after appetitive PER conditioning in which color was associated with sucrose reward. In addition, to gain insight into the neural basis of visual orientation learning, we investigated the effects of blocking OA signals in the brain. OA is a well-known neuromodulator of appetitive olfactory or visual learning in insects, including honey bees (Hammer and Menzel 1998; Mancini et al. 2018), *Drosophila* (Schwaerzel et al. 2003; Kim et al. 2013), and crickets (Unoki et al. 2005, 2006). An OA signal is crucial for mediating reward information in classical olfactory PER conditioning in honey bees (Hammer and Menzel 1998). We thus aimed at determining if it has a similar role in the experimental context of visual learning in our flight simulator.

## Materials and methods

### Experimental animals

Honey bees, *Apis mellifera* L., used in this study were reared in outdoor hives at the campus of Kobe University, Kobe, Japan. One day before the experiment, foragers with pollen loads were collected at the hive entrance and anesthetized on ice for 10 min. The hair on the mesonotum of the bee was gently shaved using a small piece of razor blade, and a small metal plate (height 10.8 × width 1.3 × thickness 0.1 mm) was fixed with a small amount of melted beeswax. The necks of bees were not fixed. Moreover, we purposely removed the pollen loads on the legs to eliminate possible disturbances in balance control during flight. Thereafter, the bees were placed in a dark incubator at 27 °C to recover from anesthesia, and after recovery, they were fed 50 µL of 30% sucrose solution. After feeding, individual bees were kept in an incubator overnight until the beginning of the experiment on the following day. The next day, we checked whether the bees exhibited normal wing beats by raising them from the ground. Bees that did not exhibit normal beating behavior were not used in the experiments.

### Flight simulator

Experiments were performed using a custom-made flight simulator (Fig. S1) constructed in a dark box. The tethered bee was mounted by attaching a metal plate on the mesonotum to a high-sensitivity torque meter (SH-02S; SUZUKO, Yokohama, Japan), and the yaw torque generated by the tethered bee was measured. We let the bee hold a small piece of paper so that it could not start flying except during the test period (see below). A 27-inch LCD monitor (ProLite G2773HS, Iiyama, Tokyo, Japan; 120 Hz refresh rate) was placed 15 cm from the bee’s head to display the visual stimuli (Fig. S1). The torque signals were fed back to the horizontal positions of the visual stimuli via a computer as they moved in the counter direction at a speed of 240-pixel sec^−1^/10^−8^ Nm. When the torque exceeded 15 × 10^−8^ Nm, the movement was at a constant speed of 3600-pixel sec^−1^. The temperature and humidity in the flight simulator were maintained at 27 ± 3 °C and 45 ± 20%, respectively, during the experiments.

### Visual stimuli

Two rectangles of different colors, blue (RGB: 0, 0, 255; dominant wavelength of 453 nm) and green (RGB: 0, 227, 0; dominant wavelength of 538 nm), on a black background (RGB: 0, 0, 0) were used for visual discriminatory learning (Fig. S2). The brightness of the two stimuli was adjusted to a fixed photon flux density [0.40 µmol m^−2^ s^−1^ measured by a spectrometer (MK350S, UPRtek, Miaoli, Taiwan)] to prevent the bee from discriminating them using intensity differences. The size of the stimuli was 32.4 (width) × 40.6° (height), as seen by the tethered bee. These visual angles ensured that bees engage their chromatic vision to achieve visual discrimination (Giurfa et al. 1996).

### Experimental procedure

**Fig. 1:**
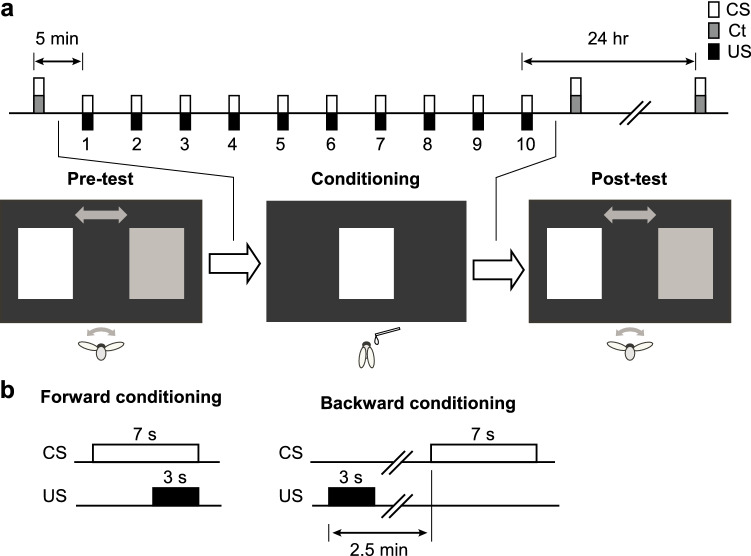
Procedure of the conditioning and tests. **a** The initial preference to the green and blue stimuli was investigated under the tethered flying condition (**Pre-test**). 5 min after the pre-test, the bee not flying received 10 times of PER conditioning trials with 5 min inter-trial intervals (**Conditioning**), in which the stimulus with less preference in the pre-test (**CS**) was presented to the bee with the sucrose reward (**US**) and the other control stimulus (**Ct**) was not presented. The color preferences were tested again 5 min and 24 h after the conditioning as tested in the pre-test (**Post-test**). **b** Time sequences of the conditioning trials. In the forward conditioning, CS and US were presented together, whereas they were separated in the backward conditioning

The experiment was performed in three steps: pre-test, conditioning, and post-test (Fig. 1a). In the pre-test, the centers of the blue and green stimuli were displayed at − 54.6° and + 54.6° from the bee, respectively, immediately after the bee was allowed to fly by removing the holding paper. The flight behavior of the bee was observed for 8 s, and the horizontal position of each visual stimulus on the monitor was recorded during flight to analyze the heading orientation of the bee with respect to each stimulus.

Five minutes after the pre-test, the bee was conditioned to associate one of the stimuli (conditioned stimulus, CS) with a reward of 30% sucrose solution (unconditioned stimulus, US), using the PER paradigm. The visual stimulus for which the bee did not exhibit preferential orientation in the pre-test was paired with a sucrose solution during training to avoid the potential effect of an initial preference bias on learning performance. During a conditioning trial, we allowed the bee to hold a small piece of paper and presented the reward stimulus (CS) at the center of the monitor for 7 s. Four seconds after the onset of the CS, sucrose solution (US) was delivered for 3 s to the antennae and then to the mouthparts of the bee using a toothpick (Fig. 1b, forward conditioning). Conditioning trials were repeated 10 times with 5 min inter-trial intervals. A control group of bees was subjected to 3 s US and 7 s CS separately 10 times at 2.5 min intervals (Fig. 1b, backward conditioning). Only individuals that exhibited PER to the US during all ten conditioning trials were used for the experiments.

Two post-tests were performed, during which the orientation toward the blue and green stimuli was recorded again in the absence of reinforcement for 8 s. One post-test was performed 5 min after the last conditioning trial, whereas the second post-test was performed 24 h later (Fig. 1a).

We only used data from bees that flew successfully for 8 s within three attempts in all tests (one pre- and two post-tests).

### Pharmacology

We conducted pharmacological experiments to clarify whether OA signals were involved in visual orientation learning in our paradigm. Epinastine hydrochloride (E5156, Sigma-Aldrich, St. Louis, MO, USA), an OA receptor antagonist, was injected into the bee brain to block OA signaling in the brain. Epinastine was dissolved in phosphate-buffered saline (PBS; P4417, Sigma–Aldrich, St. Louis, MO, USA) to obtain fresh 4 or 0.4 mM epinastine solutions before the experiments. PBS was used as a control. All injections were administered 30 min before the start of the pre-test, as in previous studies (Tedjakumala et al. 2013; Vergoz et al. 2007; Baracchi et al. 2020). The head of the unanesthetized bee that was prepared the previous day was fixed by passing its neck through the slit of a thin plastic plate. Then, the median ocellus was gently removed, and 0.2 μL of either solution was injected into the bee brain via the ocellar tract using a 10 µL syringe (701N, Hamilton, Reno, NV, USA) with a 27 G × 3/4 needle (NN-2719S, Terumo, Tokyo, Japan). After injection, the bees were kept in a dark incubator at 27 °C until the beginning of the experiment.

### Data analysis and statistics

We defined a bee as oriented to the visual stimulus when the horizontal position of the center of the stimulus was within ± 31.8° from the bee’s head. The time spent orienting toward a given stimulus during a test was calculated by summing the time of all orienting events during the test. Orientation preference was calculated as a preference index (PI), using the following equation:

$${\text{PI}}_{{\text{a}}} = \, \left( {T_{{\text{a}}} - \, T_{{\text{b}}} } \right) \, / \, \left( {T_{{\text{a}}} + \, T_{{\text{b}}} } \right),$$

where PI_a_, *T*_a_, and *T*_b_ are the PI for stimulus a, the time when the bee is oriented to stimulus a, and the time when the bee is oriented to stimulus b, respectively.

We also calculated the total time during which the bee oriented to either the blue or green stimulus to confirm if conditioning increased orientation per se toward the visual target regardless of a relationship between a rectangle color and its value (rewarded or not-rewarded), i.e.:

$${\text{Total orientation time }} = \, T_{{\text{a}}} + \, T_{{\text{b}}} .$$

The variation in the PI between the pre- and post-tests within a group was statistically analyzed using Friedman tests with Steel–Dwass post-hoc contrasts. Mann–Whitney *U* tests were used to compare PIs between the forward and backward condition groups (Fig. 3a) and Kruskal–Wallis tests were used for comparing among the PBS-, 0.4 mM epinastine- and 4 mM epinastine-injected groups (Fig. 5a). To confirm whether epinastine injection affected flying behavior, the number of bees that flew during 8-s at the first attempt in all pre- and post-tests was compared between the epinastine- and PBS-injected groups using Fisher’s exact test. All statistical tests were performed in the R environment (R Core Team 2021).

## Results

### Visual-orientation learning in tethered bees

**Fig. 2:**
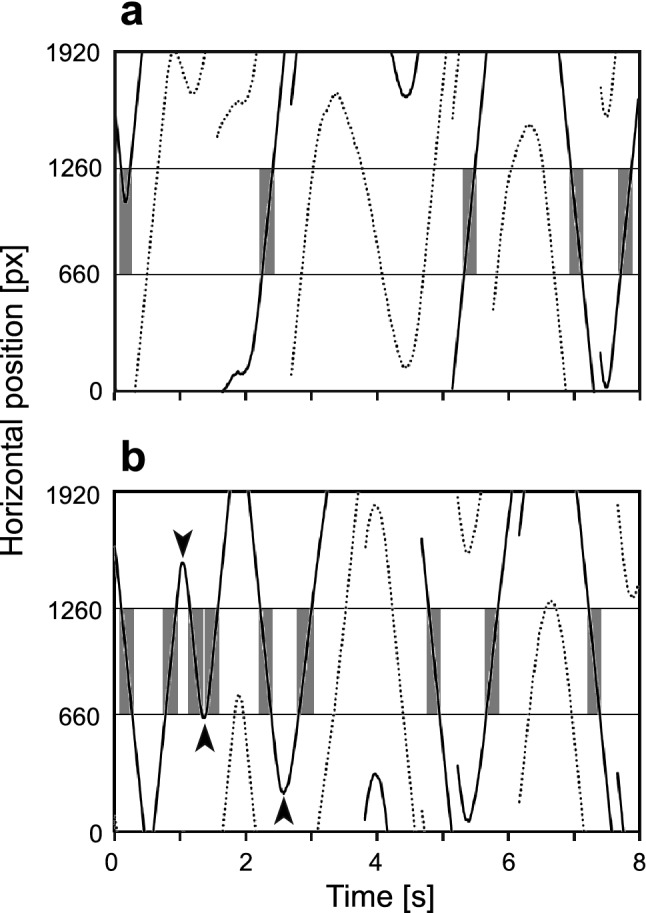
Time courses of the bee’s orientation in the pre- (**a**) and 5 min post- tests (**b**). The horizontal positions of the green (CS, solid line) and blue (Ct, dotted line) stimuli during the 8-s flight are shown. We defined the bee oriented to the stimulus when it was between the two gray horizontal lines (660–1260 px; gray bar). In the post-test, the bee often showed quick turns to CS (arrowheads). Note that the stimuli sometimes disappeared, because of the torque changes being too quick

**Fig. 3:**
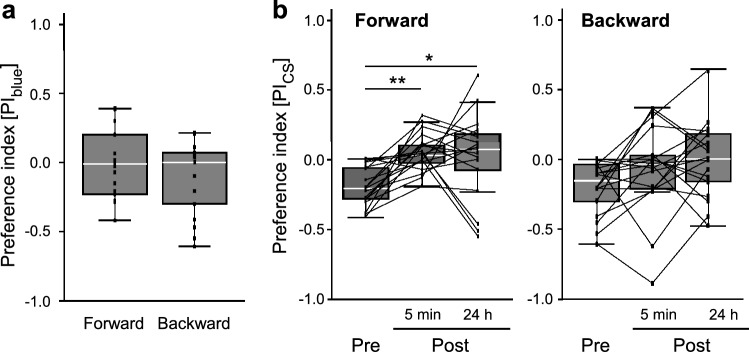
Changes in the color preferences after the conditions. **a** Initial color preferences in the forward- (*N* = 18) and the backward-conditioned (*N* = 18) groups are shown as PI_blue_ in the pre-test. There is no significant difference between two groups (*p* > 0.05, Mann–Whitney *U* test). **b** Color preferences in the forward- (*N* = 18) and backward-conditioned (*N* = 18) groups before and after the conditioning. PI_cs_ in the pre-test (**Pre**) and the post-tests, 5 min (**5 min**) and 24 h (**24 h**) after the conditioning, are shown. The PI_cs_ significantly increased after the conditioning (*p < 0.05; **p < 0.01, Friedman test with Steel–Dwass post hoc test). All box plots are shown with median (white line), the upper and the lower quartiles. Whiskers indicate 95–99% confidence intervals. Each dot indicates the PI in each tested bee and the dots connected with a line represent the data from an identical bee

Under our experimental conditions, almost all bees (96%) showed 8-s flights in all three tests (one pre- and two post-tests) in the flight simulator. Each bee received ten conditioning trials, but none of the bees showed any PER during the CS presentation throughout the conditioning period. Under the tethered condition, most bees could not fly for a long time and usually stopped flying within 1 min; therefore, we focused only on the first 8 s of the tests to investigate the initial flight orientation of each bee. A typical example of the flying orientation of a tethered bee, as indicated by the horizontal position of the stimuli in the pre- and post-test, 5 min after forward conditioning, is shown in Fig. 2. In the pre-test, the bee showed a tendency to maintain its flight direction to the right or left for a relatively long time and did not show any distinct orientation to either stimulus (Fig. 2a), suggesting an absence of strong initial visual preference. The PI values in the pre-test confirmed the absence of preference for the two visual stimuli as the median PI_blue_ in the pre-test was close to zero both in the forward and backward conditioning groups. No significant difference was found between the PI_blue_ of the two groups (Fig. 3a; *p* = 0.6576, Mann–Whitney *U* test, *N* = 18 for each group), suggesting that the experimental bees showed no orientation tendencies to either blue or green prior to conditioning. In contrast, in the 5 min post-test, the bees were more oriented toward the previously rewarded CS than in the pre-test; that is, they returned quickly to that CS after passing over it and kept it fixed in the frontal region (Fig. 2b, arrowhead). Consequently, the number of events in which the bee was oriented to the CS increased compared to that in the pre-test (Fig. 2, gray).

**Fig. 4:**
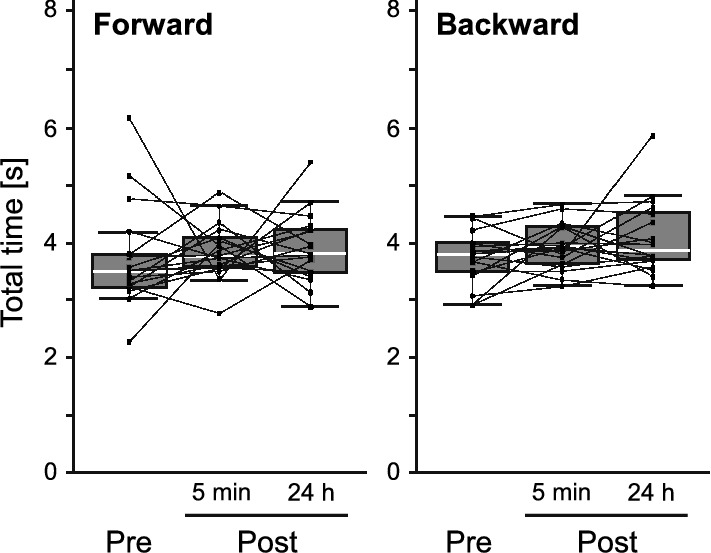
Changes in the total orientation time after the conditions. Box plots of the total times in the forward- (*N* = 18) and backward-conditioned (*N* = 18) groups are shown with median (white line), the upper and the lower quartiles. Each dot connected with a line indicates the total time in each tested bee. Whiskers indicate 95–99% confidence intervals. **Pre**, the pre-test; **5 min**, 5 min post-test; **24 h**, 24 h post-test

Moreover, behavioral changes observed following forward conditioning affected the PI_CS_ in each bee (Fig. 3b, forward). In most bees, the PI_CS_ increased between the pre-test and the 5-min post-test. A higher PI_CS_ was also observed in many bees, even 24 h post-conditioning, although some bees showed considerable decreases between the two post-tests. The PI_CS_ values were significantly different among the tests (*χ*^2^ = 15.972, d*f* = 2, *p* = 0.0003, Friedman test) as they increased from the pre-test to the 5-min and the 24-h post-tests (5 min: *p* = 0.0001; 24 h: *p* = 0.0122, Steel–Dwass test). However, in the backward-conditioned group, the PI_CS_ showed no significant changes among the three tests (Fig. 3b, backward; *χ*^2^ = 2.7324, d*f* = 2, *p* = 0.2551, Friedman test). These results indicate that the bees changed their behavioral preference after appetitive PER conditioning and oriented more toward the CS during their flight; in other words, the bees could learn to orient to the visual stimulus associated with sucrose reward. The fact that a higher PI_CS_ was maintained even after 24 h post-conditioning strongly suggests that long-term memory was established by conditioning. In contrast to the PI_CS_, the total orientation time to the two stimuli was not affected by conditioning (Fig. 4). The total orientation time showed no significant changes between the pre-test and the 5-min and 24-h post-tests in both the forward- and backward-conditioned groups (forward: pre vs. 5 min, *p* = 0.1846; pre vs. 24 h, *p* = 0.320; 5 min vs. 24 h, *p* = 0.9890; backward: pre vs. 5 min, *p* = 0.4997; pre vs. 24 h, *p* = 0.2970; 5 min vs. 24 h, *p* = 0.8684, Steel–Dwass test). Therefore, we concluded that the increase in the PI_CS_ after forward conditioning was not due to the enhancement of the orientation to the visual targets but to the increase in relative orienting time to CS.

### Effect of blockade of OA signal on learning

**Fig. 5:**
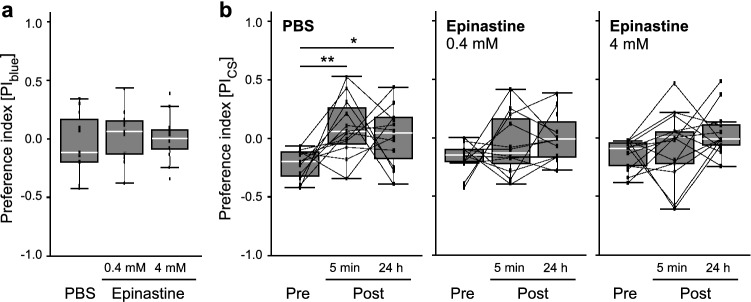
Effects of the epinastine injection on the visual orientation learning. **a** Initial color preferences in the PBS- and epinastine- (0.4 or 4 mM) injected groups are shown as PI_blue_ in the pre-test. There is no significant difference among the PBS- (*N* = 12) and the two epinastine-injected groups (*N* = 12, 0.4 mM; *N* = 13, 4 mM; *p* > 0.05, Kruskal–Wallis test). **b** Color preferences in the PBS- (*N* = 12), 0.4 mM epinastine- (*N* = 12) and 4 mM epinastine- (*N* = 13) injected groups before and after the conditioning. PI_cs_ in the pre-test (**Pre**) and the post-tests, 5 min (**5 min**) and 24 h (**24 h**) after the conditioning, are shown. The PI_cs_ significantly increased after the conditioning only in the PBS-injected group (*p < 0.05; **p < 0.01, Friedman test with Steel–Dwass post hoc test). All box plots are shown with median (white line), the upper and the lower quartiles. Whiskers indicate 95–99% confidence intervals. Each dot indicates the PI in each tested bee and the dots connected with a line represent the data from an identical bee

To examine the possible role of OA signaling in visual orientation learning in bees, epinastine, an OA receptor antagonist, was injected into the brain before the pre-test. Epinastine injection caused no serious behavioral changes in the tethered bees. However, some bees showed relatively shorter flights compared with the non-injected and PBS-injected bees; consequently, two or three attempts were needed to obtain 8-s flight data in the test. In the 4 mM epinastine-injected group, significantly fewer bees (5 out of 13) succeeded in an 8-s flight on the first attempt in all three tests compared to those in the PBS-injected group (12 of 12) (*p* = 0.0001, Fisher’s exact test), whereas we could not find any significance between the 0.4 mM epinastine-injected (10 of 12) and PBS-injected groups (*p* > 0.1, Fisher’s exact test). The PI_blue_ in the pre-test was not affected by the injection (Fig. 5a). As in the non-injected bees (see Fig. 3a), the median of the PI_blue_ was close to zero in all groups and no significant differences in the PI_blue_ among groups were found (*χ*^2^ = 3.5576, d*f* = 2, *p* = 0.1668, Kruskal–Wallis test, *N* = 12/12/13 in PBS/0.4 mM/4 mM group), reiterating the fact that the bees did not have any initial preferences for green or for blue before the conditioning.

**Fig. 6:**
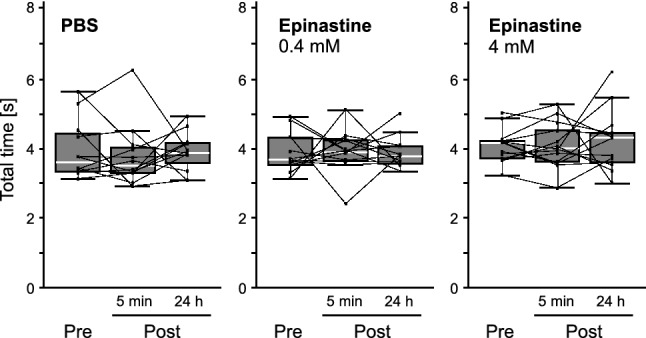
Effects of the epinastine injection on the total orientation time. Box plots of the total times in the PBS- (*N* = 12), 0.4 mM epinastine- (*N* = 12) and 4 mM epinastine- (*N* = 13) injected groups are shown with median (white line), the upper and the lower quartiles. Each dot connected with a line indicates the total time in each tested bee. Whiskers indicate 95–99% confidence intervals. **Pre**, the pre-test; **5 min**, 5 min post-test; **24 h**, 24 h post-test

Similar to the non-injected bees, the bees showed significant differences in PI_CS_ among the three tests after PBS injection (Fig. 5b, PBS; *χ*^2^ = 8.1667, d*f* = 2, *p* = 0.0169, Friedman test) due to a significant increase from pre-test to the 5-min and the 24-h post-tests (5 min: *p* = 0.0029; 24 h: *p* = 0.0298, Steel–Dwass test), whereas no significant changes were observed in the total orientation time among the tests (Fig. 6, PBS; pre vs. 5 min, *p* = 0.7333; pre vs. 24 h, *p* = 0.9020; 5 min vs. 24 h, *p* = 0.5163, Steel–Dwass test). In contrast, epinastine-injected bees exhibited impaired visual orientation learning. In both low- and high-dose epinastine groups, there were no significant changes in the PI_CS_ among the three tests (Fig. 5b, epinastine; 0.4 mM: *χ*^2^ = 1.1667, df = 2, *p* = 0.5580; 4 mM: *χ*^2^ = 4.7692, d*f* = 2, *p* = 0.092, Friedman test). The total orientation time to the two stimuli in the epinastine-injected groups did not differ significantly among tests (Fig. 6, Epinastine; 0.4 mM: pre vs. 5 min, *p* = 0.9018; pre vs. 24 h, *p* = 0.9710; 5 min vs. 24 h, *p* = 0.7333; 4 mM: pre vs. 5 min, *p* = 0.9943; pre vs. 24 h, *p* = 0.9320; 5 min vs. 24 h, *p* = 0.9911, Steel–Dwass test). Taken together, these results suggest that the OA in the brain may play a role in visual orientation learning.

## Discussion

### Orientation behavior in the flight simulator

Our flight simulator revealed that tethered bees oriented to the learned color stimulus after appetitive conditioning. Additionally, our study indicates that heading to a particular stimulus and fixation time for the choice experiment in the VR setup are reliable indices for assessing the bees’ orientation behavior in both flying and walking bees (Buatois et al. 2017, 2018; Lafon et al. 2021, 2022; Geng et al. 2022).

Considering the flight velocity for a foraging flight of bees (Menzel et al. 2005), the 8-s flight that was measured during the experiment can be estimated as a 42 m flight, which is a much shorter flight than that undertaken in natural foraging (Visscher and Seeley 1982). Therefore, the orientation behavior analyzed must be a steering behavior at the very early part of the navigation, as revealed by Menzel et al. (2011) who reported an initial straight flight observed immediately after departure from the hive. Subsequently, bees would use other environmental cues, such as landmarks, geometrical features, polarized light, and memory, to reach their target location. Otherwise, the observed behavior might be a scanning behavior against the visual stimulus that honey bees desire to reach during and after learning. Nevertheless, our results showed that appetitive conditioning enables honey bees to orient themselves to a learned stimulus.

Compared to findings in *Drosophila* (Liu et al. 2006), the period of one orientation event in honey bees did not change, but the number of events increased (gray bars in Fig. 2). Time courses of the horizontal position of the CS show that learned honey bees often pass over the CS but quickly returned to it (arrowheads in Fig. 2). At present, it is unclear whether this behavior is a species-specific property of the visual orientation of honey bees. Another possibility is that the moving velocity of the visual stimuli may be faster than the steering reaction of the honey bee. If this is the case, then the feedback signal must be appropriately tuned.

In addition to olfactory learning, harnessed honey bees showed visual learning after appetitive PER conditioning (e.g., color: Niggebrügge et al. 2009; Hori et al. 2006; Lichtenstein et al. 2019, motion direction: Hori et al. 2007, and polarized light e-vector orientation: Sakura et al. 2012). Visual learning in harnessed bees generally requires more training trials and results in poorer learning performance compared to that in free-flying bees (see review by Avarguès-Weber and Mota 2016). In the present study, bees exhibited no PER to the CS during the training trials. It is currently difficult to provide a convincing explanation for this observation. The fixed situation of honey bees in previous studies (mostly mounted in a metal or plastic tube) differed from that in our study (hung from the torque meter). We speculate that this might have caused differences in PER sensitivity to US. Indeed, hung bees rarely showed PER to 30% sucrose solution if bees were collected 2–3 h before PER treatment (personal observation). Nevertheless, we confirmed that the bees learned the CS when it was paired with US as shown by the flight orientation to CS in the post-tests. Moreover, the established memory was maintained even at 24 h post-conditioning. Flight orientation could be a good criterion for evaluating visual memory given that it is often linked to the location of food.

### Role of OA in learning

We found that epinastine impaired visual orientation learning (Fig. 5). This is consistent with the fact that OA signaling is required for appetitive learning in ants (Wissink and Nehring 2021), *Drosophila* (Kim et al. 2013), crickets (Mizunami and Matsumoto 2017), and honey bees (Mancini et al. 2018). In our study, two concentrations (4 mM and 0.4 mM) of epinastine, which antagonize mainly one type of honey bee OA receptor (AmOA1, also known as AmOctα1R), significantly reduced the PI_CS_ post-conditioning, and these two concentrations had the same effect (Fig. 5). Although 4 mM epinastine may be considered higher than the physiological concentration, it is commonly used in honey bees (Tedjakumala et al. 2013; Rusch et al. 2021; Baracchi et al. 2020; Vieira et al. 2018). Thus, serious functional deficits should not occur, except for an antagonistic effect against OA receptors. The total orientation time during flight in the treated group was not significantly different from that in the control group (Fig. 6).

However, one cannot exclude the possibility that high epinastine concentrations may result in other biological effects, such as a decrease in motivation. For example, blockade of OA signals might influence the flight activity of bees. In *Drosophila*, the inhibition of octopaminergic neurons reduces flight duration (Brembs et al. 2007; Manjila et al. 2019). Our experiments revealed that the 4 mM epinastine-treated bees required significantly more attempts to obtain an 8-s flight, whereas the PBS-treated bees did not (one attempt in all cases). This difference in the number of attempts required between the two groups might have resulted from the epinastine treatment.

Although epinastine exhibits high specificity in blocking OA signaling via AmOA1, epinastine also acts to a minor extent as an antagonist of DA receptors (AmDOP2, Beggs et al. 2011) and tyramine (TA) receptors (AmTAR2, Reim et al. 2017). This indicates that epinastine may impair DA and TA signaling, although the blocking efficiency of epinastine for AmDOP2 and AmTAR2 is much weaker than that for AmOA1. Recent molecular biology studies have found other types (AmOAα2R and AmOARβ1-3) of OA receptors (Balfanz et al. 2014; Blenau et al. 2020). However, the affinity of these receptors to epinastine is unclear. All types of OA receptors exhibited OA-induced changes in cAMP levels in cultured cells upon application of OA or TA, although the EC50 of TA was at least one order of magnitude higher than that of OA (Grohmann et al. 2003; Balfanz et al. 2014; Blenau et al. 2020). However, as Grohmann et al. (2003) concluded, the increase in intracellular cAMP concentration observed at high OA concentrations may be a secondary effect, induced by massive Ca^2+^ release. In addition to OA, DA is related to appetitive learning in *Drosophila* (Yamagata et al. 2015) and ants (Wissink and Nehring 2021). Thus, we do not exclude any potential contribution of these biological amines to visual orientation learning. Comprehensive pharmacological experiments are necessary to confirm the role of OA in visual orientation learning, that is, the application of OA, DA, and TA antagonists, such as mianserine and fluphenazine, and agonists, such as chlordimeform, at different injection times.

### Neural basis of navigation in insects

The mushroom body is a crucial neuropil for visual learning in insects, as well as olfactory learning (*Drosophila*: Vogt et al. 2014; cockroach: Mizunami et al. 1998; honey bee: Plath et al. 2017; ant: Buehlmann et al. 2020). An octopaminergic ventral unpaired median neuron 1 of the maxillary neuromere, VUMmx1, responds to sucrose solution and projects onto the mushroom body (Kreissl et al 1994; Menzel 2012). Therefore, VUMmx1 transmits a reward signal during classical olfactory conditioning. A detailed anatomical study showed that VUMmx1 projects to the lip and basal ring of the mushroom body calyx (Schröter et al 2007). The basal ring also receives visual information directly from the medulla and lobula (Mobbs 1984; Gronenberg 1986; Ehmer and Gronengerg 2002). Thus, color (CS) and reward (US) information is likely to be integrated first in the basal ring. Immunostaining for AmOA1 revealed positive signals in the basal ring (Sinakevitch et al. 2011). Because both AmOA1-immunoreactive class I and II Kenyon cells have dendrites in this region (Sinakevitch et al. 2011), determining which subpopulations of the mushroom body are involved in color orientation learning is not possible. Further experiments, such as local blockade of OA signals in a mushroom body subpopulation (Buehlmann 2020; Plath et al. 2017), are needed to draw better information flow from the optic lobe to higher order brain regions.

In addition, strong AmOA1-immunoreactive signals have been found in some neurons in the anterior superior optical tract (asot) that connects the medulla to the calyx (Sinakevitch et al. 2011). Some AmOA1-immunoreactive asot neurons may receive OA-mediated reward signals. Mushroom body feedback neurons in the protocerebral-calycal tract (PCT) are also AmOA1 positive (Sinakevitch et al. 2011). PCT neurons are GABAergic neurons involved in olfactory learning (Okada et al. 2007). Taken together, neural plasticity underlying color orientation learning may occur in either or all of the (1) asot neurons, (2) Kenyon cells, and (3) PCT neurons. In any case, mushroom body output neurons should convey learning-related signals to other areas of the brain (Paffhausen et al. 2020).

Another higher order center in the insect brain is the central complex, which is involved in steering behavior (Guo and Ritzmann 2013; Stone et al. 2017; Heinze 2017) and place learning (Ofstad et al. 2011; Varga et al. 2017). Local inactivation by procaine suggests different roles for the mushroom body and the central complex in honey bee aversive color learning. The mushroom body is involved in the integration of US and CS and memory formation, and the central complex initiates escape from danger (Plath et al. 2017). Additionally, the anatomical connectome revealed direct and indirect connections between the mushroom body and central complex in *Drosophila* (Li et al. 2020; Hulse et al. 2021). Taken together, the mushroom body and the central complex may coordinate visual orientation learning with different roles (Collett and Collett 2018; Heinze 2017). For instance, the mushroom body is involved in ‘where’ to go, and the central complex is involved in ‘how’ to go there.

## Supplementary Information

Below is the link to the electronic supplementary material.

Supplementary file1 (PDF 103 KB)

## Data Availability

The datasets generated and/or analyzed during the current study are available from the corresponding authors upon reasonable request.

## Abbreviations

CS: Conditioned stimulus
OA: Octopamine
PI: Preference index
US: Unconditioned stimulus
