# Supplementary material for: Visual learning in tethered bees modifies flight orientation and is impaired by epinastine
Source: J Comp Physiol A Neuroethol Sens Neural Behav Physiol. 2023 Mar 17;209(4):529–39. doi: 10.1007/s00359-023-01623-z (PMC10354131; doi:10.1007/s00359-023-01623-z)
Supplement: Supplementary file 1 — Supplementary file1 (PDF 103 KB) [file 359_2023_1623_MOESM1_ESM.pdf]

Journal of Comparative Physiology A

Visual learning in tethered bees modifies flight orientation and is impaired by epinastine

Kobayashi N, Hasegawa Y, Okada R, Sakura M
Department of Biology, Graduate School of Science, Kobe University
skr@port.kobe-u.ac.jp (Midori Sakura)

Supplementary Figures

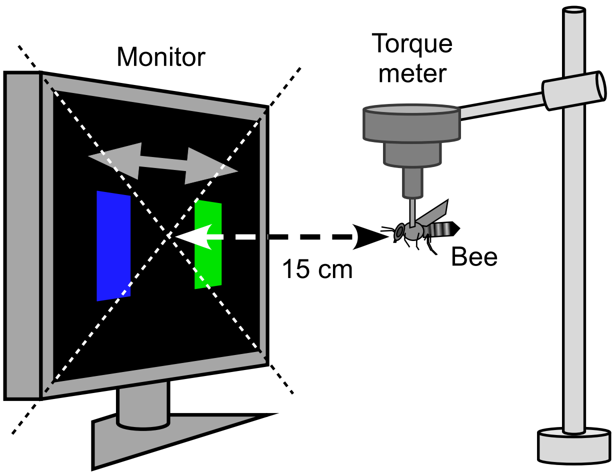

Fig. S1 Experimental setup. A bee was tethered to a torque meter by a metal plate attached to the mesonotum and positioned in front of the center of a computer monitor, on which the blue and green rectangle images were displayed. The distance between the monitor and the bee's head was 15 cm. The yaw torque generated by the bee during the flight was recorded and translated into the horizontal movements of the visual stimuli to the counter direction.

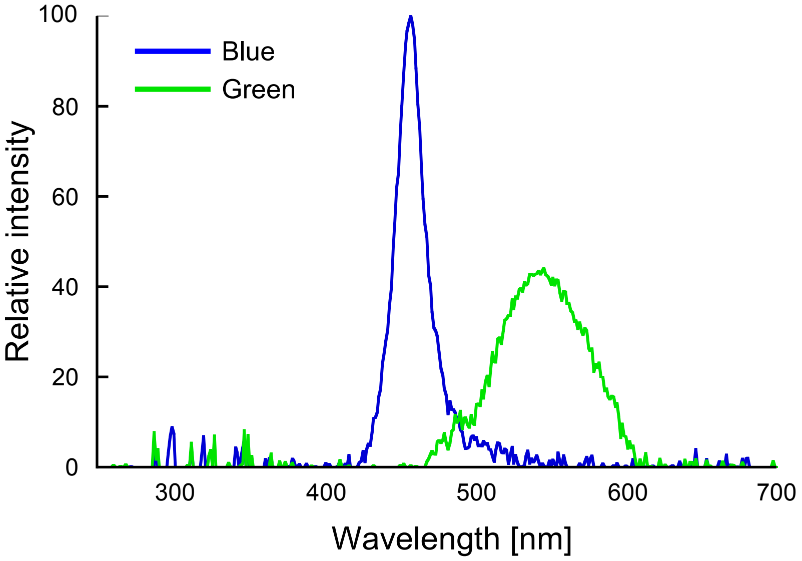

Fig. S2 Color spectra of the blue (dominant wavelength = 453 nm) and green (dominant wavelength = 538 nm) stimuli used for the experiments. Relative intensities of each stimulus displayed on the PC monitor were measured using a spectrometer (UnispeKs, UNISOKU Scientific Instruments, Osaka, Japan) from the tethered bee location. The brightness of two stimuli was adjusted with the same photon flux density (0.40 µmol $m^{-2}s^{-1}$).
